# Light-Dependent Temporal Reprogramming of Alternative Splicing Dynamics Under Salt Stress in Sweet Potato (*Ipomoea batatas* [L]. Lam)

**DOI:** 10.3390/plants15101556

**Published:** 2026-05-20

**Authors:** Yuanru Luo, Feiyan Gao, Huifeng Luo, Lipeng Gao, Yu Wang, Mengzhao Wang, Tianjia Liu, Yongping Li, Guopeng Zhu

**Affiliations:** 1 School of Breeding and Multiplication (Sanya Institute of Breeding and Multiplication), Hainan University, Sanya 572025, China; 24210902000010@hainanu.edu.cn (Y.L.); 23220951310226@hainanu.edu.cn (F.G.); glp2000nsd@163.com (L.G.); 17709603697@163.com (Y.W.); mzwang@hainanu.edu.cn (M.W.); tanja1010@hainanu.edu.cn (T.L.); 2 Key Laboratory for Quality Regulation of Tropical Horticultural Crops of Hainan Province, School of Tropical Agriculture and Forestry, Hainan University, Haikou 570228, China; 3 Institute of Horticulture, Hangzhou Academy of Agricultural Sciences, Hangzhou 310024, China; huifengluo@163.com

**Keywords:** alternative splicing, light signaling, RNA-seq, salt stress, sweet potato

## Abstract

Soil salinity is a major constraint on crop productivity, and plants rely on multilayered regulatory mechanisms to adapt to stress. Alternative splicing (AS) enhances transcriptome plasticity, yet how light modulates AS under salt stress remains unclear. Here, we performed a transcriptome-wide analysis to investigate light-dependent AS dynamics in sweet potato under salt stress. Plant treatments were initiated during daytime (SD) and nighttime (SN) conditions, and samples were collected at five time points (0–8 h). Intron retention (IR) was the predominant AS type (~36–37%), followed by A3SS, A5SS, and exon skipping (SE). Notably, light enhanced both the magnitude and temporal dynamic of AS, with a pronounced early response (0–2 h) under SD, where differential AS (DAS) events were nearly doubled compared with SN. This early AS response was accompanied by an increased prevalence of IR events and upregulation of spliceosome-related genes, suggesting dynamic splicing regulation under light. Enrichment of the mRNA surveillance pathway further indicates that IR-derived transcripts may be subject to RNA quality control. Although enriched pathways were largely conserved between SD and SN, including spliceosome and mRNA surveillance, more DAS genes under SD indicate enhanced responsiveness of conserved regulatory networks. These findings demonstrate that light reshapes the temporal dynamics of AS under salt stress, primarily through IR and its coupling with RNA surveillance, providing new insights into post-transcriptional regulation in crop stress adaptation.

## 1. Introduction

Soil salinization has become an increasingly severe global agricultural constraint, substantially limiting crop productivity and sustainability. It is estimated that more than 20% of irrigated agricultural land worldwide is affected by salinity, and this proportion is expected to further increase due to climate change, excessive irrigation, and inappropriate land management practices [1]. Salt stress imposes complex adverse effects on plants through osmotic stress, ionic toxicity, and secondary stresses, thereby disrupting essential physiological processes such as photosynthesis, metabolism, and signal transduction [2,3,4]. Consequently, excessive salinity inhibits seed germination, delays vegetative growth, interferes with tissue and organ differentiation, induces dwarf phenotypes, and ultimately leads to significant yield losses [5,6]. To cope with these challenges, plants have evolved multilayered adaptive mechanisms, including ion homeostasis, osmotic adjustment, antioxidant defense, and transcriptional reprogramming [5,7,8]. In addition to transcriptional control, post-transcriptional regulation has emerged as a critical layer in stress adaptation, enabling rapid and dynamic modulation of gene function without altering gene expression levels. Among these mechanisms, alternative splicing (AS) plays a central role in reshaping transcriptome complexity under stress conditions.

Alternative splicing enables a single gene to generate multiple transcript isoforms through differential splice site selection, thereby expanding transcriptomic and proteomic diversity [9]. AS allows a single gene to generate multiple transcript isoforms through differential splice site selection, thereby greatly expanding transcriptomic and proteomic diversity. Four primary types of AS events are recognized: exon skipping (ES), intron retention (IR), alternative 5′ splice site (A5SS), and alternative 3′ splice site (A3SS). Genome-scale transcriptomic studies have shown that AS is highly prevalent in plants and is particularly responsive to abiotic stresses, including salinity [5,10,11]. Among different AS types, IR is the most abundant in plants and is frequently enhanced under salt stress conditions [12,13], functioning as a regulatory mechanism that modulates transcript stability and translation through RNA surveillance pathways [14,15]. Beyond stress signaling, light has emerged as a key environmental regulator of AS. Studies in *Arabidopsis* have demonstrated that light–dark transitions rapidly reshape AS patterns, affecting genes involved in circadian rhythms, RNA metabolism, and stress responses [16,17]. Mechanistically, light-regulated AS has been associated with RNA polymerase II elongation kinetics, splicing factor activity, and energy-dependent regulation of the spliceosome. Notably, darkness is often linked to increased intron retention, suggesting that AS decisions are closely coupled with cellular energy status and metabolic activity [17,18]. However, despite extensive evidence that both salt stress and light independently influence AS, how these signals interact to coordinately shape AS dynamics remains poorly understood, particularly in crop species under realistic light–dark cycles. Recent advances in single-cell and spatial transcriptomics have revealed substantial cell-type-specific heterogeneity in plant stress responses, which is often masked in bulk RNA-seq analyses. This limitation highlights the need to integrate high-resolution transcriptomic approaches with AS analysis to better resolve the spatial and cellular specificity of stress-responsive regulatory networks [19].

Sweet potato (*Ipomoea batatas* (L.) Lam), an important global food crop, is a vital source of calories, vitamins, and minerals, particularly in developing nations [20,21]. Despite its relatively high adaptability compared with other staple crops, sweet potato production is still severely constrained by high salinity, which negatively affects tuberization, biomass accumulation, and starch synthesis [22,23,24,25]. Although previous studies have characterized transcriptional responses to salt stress in sweet potato, the role of AS—especially under different light conditions—remains largely unexplored. In natural environments, salt stress occurs under dynamic light–dark cycles, suggesting that AS regulation may differ substantially between daytime and nighttime stress exposure. Therefore, we hypothesize that light modulates the temporal dynamics and functional specificity of salt stress-induced AS responses, leading to distinct regulatory outcomes. To test this hypothesis, we performed a transcriptome-wide analysis of AS under salt stress in both light (SD) and dark (SN) conditions across multiple time points. This study aims to (i) characterize the temporal dynamics of AS under contrasting light regimes, (ii) determine whether light alters the magnitude and type of AS events, and (iii) explore the functional implications of light-dependent AS regulation. Our findings provide new insights into how environmental signals are integrated at the post-transcriptional level to regulate stress adaptation in crops.

## 2. Results

### 2.1. Phenotypic Differences Under Salt Stress Between Light and Dark Conditions

**Figure 1:**
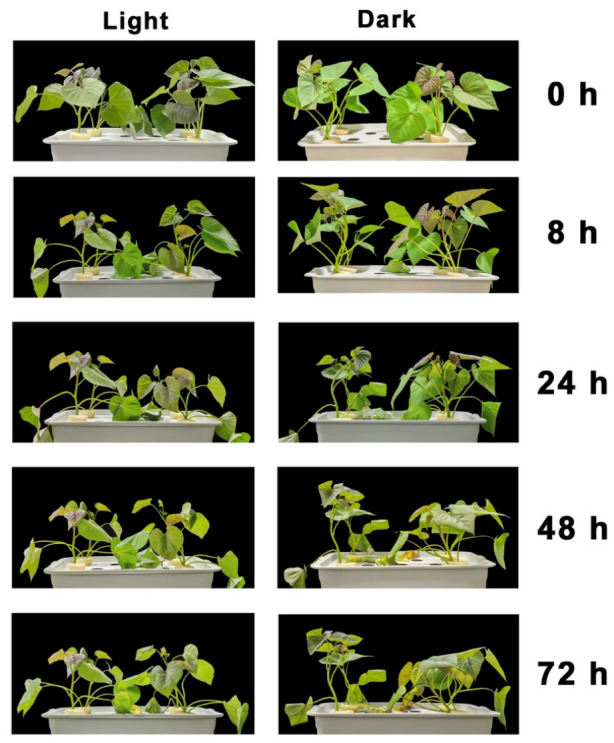
Phenotypic responses of sweet potato seedlings to salt stress under light and dark conditions. Sweet potato seedlings (cultivar ‘Haida 7791’) were subjected to 200 mM NaCl treatment under light (SD, Salt stress during the Day; **left** **panels**) and dark (SN, Salt stress during the Night; **right panels**) conditions. Representative images were taken at 0, 8, 24, 48, and 72 h after treatment initiation. The figure illustrates the progressive morphological changes, including leaf wilting, chlorosis, and growth inhibition, allowing visual comparison of stress responses between light and dark environments over time.

To investigate the effects of light conditions on the alternative splicing response to salt stress, uniform seedlings of the sweet potato variety ‘Haida 7791’ were grown under a 12 h light/12 h dark photoperiod. Salt stress (200 mM NaCl) was initiated at two different time points: during the light phase (ZT0, designated as SD) and during the dark phase (ZT16, designated as SN). Leaf tissues were collected at five time points (0, 2, 4, 6, and 8 h) after the onset of salt treatment, with three independent biological replicates at each time point, and subsequently used for RNA extraction and transcriptome sequencing (Table S1). Phenotypic evaluation revealed clear differences between the two conditions throughout the stress period: seedlings in which salt stress was initiated during the light phase maintained better leaf color and less severe chlorosis, whereas those in the SN group exhibited more pronounced growth retardation, wilting, and chlorophyll degradation (Figure 1). These results indicate that the physiological response of sweet potato to salt stress differs depending on the light phase at which stress is initiated.

### 2.2. AS Profiles in Leaves Under Salt Stress in Light and Dark Conditions

**Figure 2:**
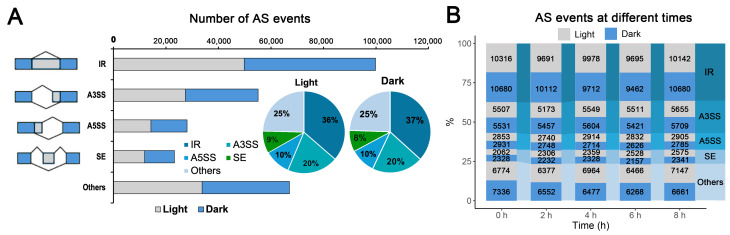
Global profiling of alternative splicing (AS) events at different time points during salt stress under light and dark conditions. (**A**) Distribution of AS events in light and dark conditions at different time points. IR: intron retention; ES: exon skipping; A3SS: alternative 3′ splice site; A5SS: alternative 5′ splice site. (**B**) Temporal dynamics of each AS event type across different time points under both conditions, showing how specific splicing patterns respond to salt stress and differ between light and dark treatments.

To investigate the dynamics of AS in response to salt stress under different light conditions, we performed comprehensive profiling of AS events across five time points (0, 2, 4, 6, and 8 h) under both light (Salt stress during the Day, SD) and dark (Salt stress during the Night, SN) conditions. Using the AStalavista tool v4.0, four major AS types (IR, A3SS, A5SS, and SE), together with other complex AS events, were identified at each time point. The results suggest that overall AS abundance remains comparable between conditions but may differentially affect specific AS types or regulatory pathways. Among the four major AS types, IR was the most abundant in both light and dark conditions (Light: 49,822, 36%; Dark: 49,971, 37%), followed by A3SS (Light: 27,395, 20%; Dark: 27,722, 20%), A5SS (Light: 14,244, 10%; Dark: 13,804, 10%), and SE (Light: 11,830, 9%; Dark: 11,367, 9%) (Figure 2A). The predominance of IR events under both conditions is consistent with previous reports in other plant species [26,27].

Temporal analysis further revealed that IR events remained the dominant AS type at all five time points in both light (SD) and dark (SN) groups, with counts ranging from 9695 to 10,316 in the light group and from 9462 to 10,142 in the dark group (Figure 2B). A3SS event counts ranged from 5173 to 5655 in the SD group and from 5421 to 5709 in the SN group. Notably, SE events were more abundant under light than dark conditions across all post-stress time points (SD: 2062–2575; SN: 2157–2341), suggesting that exon definition is more sensitive to light-dependent transcriptional regulation during salt stress. Other complex AS events ranged from approximately 6377 to 7147 in the SD group and from 6268 to 7336 in the SN group.

### 2.3. Comparison of AS Genes Between Light and Dark Conditions

**Figure 3:**
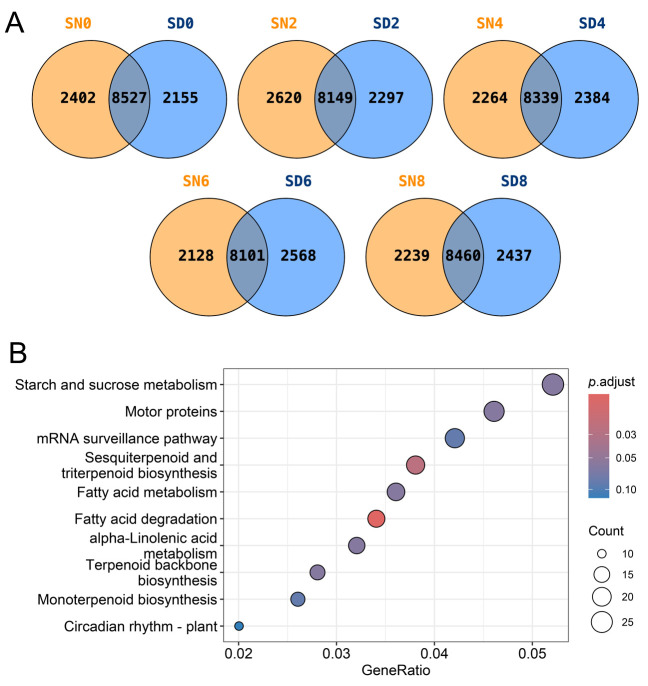
Comparative analysis of AS events at different time points under the light and dark conditions. (**A**) Venn diagram showing common and unique AS genes at different time points between the light (SD) and dark (SN) treatments across all sampled time points under salt stress. (**B**) Top 10 enriched KEGG pathways of unique AS genes in the light group.

To determine the extent to which light conditions influence the repertoire of AS genes under salt stress, the overlap and uniqueness of AS genes between SD and SN groups at each time point were analyzed using Venn diagrams (Figure 3A). This pattern of substantial shared (but also condition-specific) AS was consistently maintained throughout the experiment: overlapping gene counts ranged from 8101 to 8527 across all time points, while light-specific AS genes ranged from 2128 to 2620 and dark-specific AS genes from 2155 to 2437.

Kyoto Encyclopedia of Genes and Genomes (KEGG) enrichment analysis of the unique AS genes in the SD group revealed significant enrichment in several biologically distinct pathways (Figure 3B, Table S2). The top enriched pathways included starch and sucrose metabolism, motor proteins, mRNA surveillance pathway, sesquiterpenoid and triterpenoid biosynthesis, fatty acid metabolism, fatty acid degradation, alpha-linolenic acid metabolism, terpenoid backbone biosynthesis, monoterpenoid biosynthesis, and circadian rhythm—plant. The enrichment of the mRNA surveillance pathway among light-specific AS genes suggests that illuminated conditions may be associated with RNA quality control processes of AS-derived transcripts, potentially facilitating the degradation of non-productive isoforms generated under salt stress. The concurrent enrichment of terpenoid and fatty acid metabolism pathways indicates that light conditions may specifically orchestrate secondary metabolite production and membrane lipid remodeling as components of the salt stress adaptive response.

### 2.4. Differentially Alternatively Spliced Events Between Light and Dark Conditions

**Figure 4:**
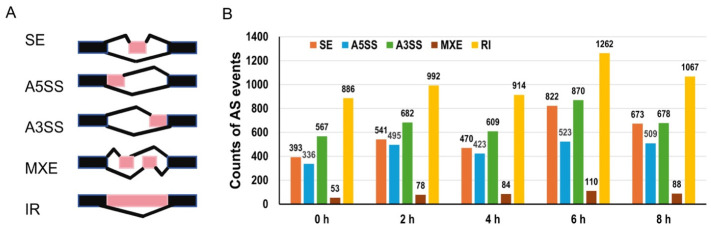
Differentially alternatively spliced (DAS) events at different time points between light and dark conditions. (**A**) Schematic representation of five AS event types detected by rMATS: A5SS (alternative 5′ splice site), A3SS (alternative 3′ splice site), MXE (mutually exclusive exons), IR (intron retention), and SE (skipped exon). Constitutive exons are shown as black boxes, alternatively spliced regions are highlighted in pink, and introns are represented by black connecting lines. (**B**) Number of differentially alternatively spliced events between light (SD) and dark (SN) conditions under salt stress at each time point.

To characterize how light conditions specifically modify salt stress-induced AS, DAS events between the SD and SN groups were identified at each time point using rMATS [28]. Five types of AS events were analyzed: SE, A5SS, A3SS, MXE, and RI (Figure 4A, Table S3). At 0 h, before salt treatment, substantial differences already existed between the two conditions, with 393 SE, 336 A5SS, 567 A3SS, 53 MXE, and a comparatively higher number of RI events between SD and SN (Figure 4B). Following salt application, the total number of DAS events between the two conditions increased progressively over time, with SE events showing a particularly marked and sustained increase throughout the stress period. This progressive divergence in SE between SD and SN groups suggests that light conditions exert an increasingly important influence on exon inclusion decisions as the salt stress response unfolds, pointing to a light-dependent regulatory layer that shapes the dynamic trajectory of exon usage during stress adaptation.

**Figure 5:**
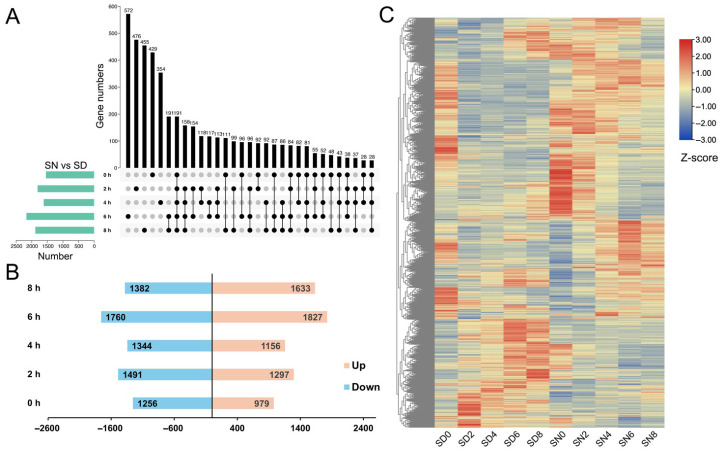
Dynamic changes in differentially alternative spliced (DAS) events under light (SD) and dark (SN) conditions. (**A**) The number of DAS events detected at each time points under light (SD) and dark (SN) conditions. (**B**) Number of DASs at five time points between light and dark conditions. (**C**) The expression trends of all DAS genes at different time points in light and dark conditions.

The UpSet plot revealed that the number of DASs varied considerably across time points, with the largest single time point group comprising 572 genes unique to a single comparison, followed by 476, 455, 429, and 354 genes in the next most abundant groups (Figure 5A). Notably, a subset of DASs was shared across multiple time points, with 191, 158, and 113 DAS events common to three or more conditions, indicating that a core set of DASs consistently exhibits differential splicing across multiple time points. Additionally, smaller intersecting groups—including sets of 191, 117, 99, 96, and 92 genes—were shared across specific combinations of time points, suggesting that distinct waves of condition-specific AS reprogramming occur at defined stages of the stress response.

We then counted the number of DASs at each time point between light and dark conditions (Figure 5B). At 0 h, 1256 DASs showed predominantly decreased exon inclusion levels, whereas 979 DASs showed predominantly increased inclusion levels. At 2 h, the number of DASs showing decreased inclusion levels increased to 1491, while 1297 showed increased inclusion levels. At 4 h post-stress, DASs showing decreased inclusion levels declined to 1344, with 1156 showing increased inclusion levels, suggesting a partial return toward a balanced distribution of exon inclusion changes. By 6 h, both categories reached their highest counts (decreased inclusion: 1760; increased inclusion: 1827), indicating a renewed and maximal divergence in DAS splicing patterns between light and dark conditions at this stage. At 8 h, DASs showing decreased and increased inclusion levels numbered 1382 and 1633, respectively. Across all time points, the number of DASs showing increased inclusion levels in dark relative to light was comparable to or exceeded that of those showing decreased inclusion levels, particularly at 6 h and 8 h, suggesting that dark conditions may broadly promote shifts in exon inclusion patterns rather than suppress alternative splicing activity during the later stages of salt stress.

To further examine the transcriptional behavior of genes associated with alternative splicing changes, we analyzed the expression patterns of genes harboring DAS events across the experimental time course. A hierarchical clustering heatmap was constructed using Z-score normalized expression values across all ten sample groups (SD0-8, SN0-8) (Figure 5C). The heatmap revealed two broad and contrasting expression clusters. One major cluster comprised genes with consistently high expression under SD conditions (particularly at SD0–SD4) and relatively low expression under SN conditions, while the other cluster showed the converse pattern, with elevated expression under SN conditions. Within each major cluster, further subclusters were discernible, reflecting more nuanced temporal expression dynamics—for example, genes preferentially expressed in the early SD time points (SD0–SD2) versus those maintained at relatively high levels throughout the SD series, and genes transiently elevated at early SN time points versus those gradually accumulating under SN across the time points. These expression patterns highlight a pronounced transcriptional divergence between light and dark conditions during salt stress. Importantly, as these genes are defined based on DAS events, the results suggest that alternative splicing regulation is accompanied by coordinated but distinct transcriptional programs under SD and SN conditions.

### 2.5. Relationship Between Alternative Splicing and Gene Expression Under Light Conditions

**Figure 6:**
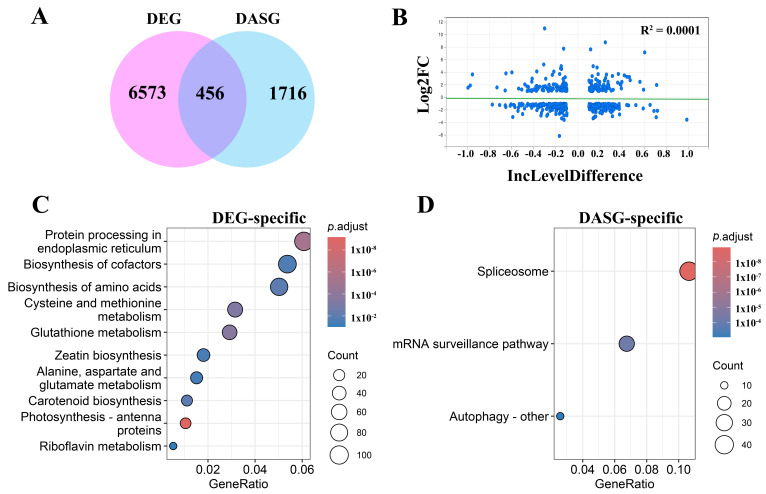
Distinct and coordinated regulation of gene expression and alternative splicing under light conditions. (**A**) Venn diagram showing the overlap between differentially expressed genes (DEGs) and genes undergoing differential alternative splicing (DASGs) under light conditions (0 h vs. 2 h). (**B**) Scatter plot showing the relationship between gene expression changes (log2FC) and alternative splicing changes (IncLevelDifference) for overlapping genes. Each blue dot represents a gene. The green horizontal line denotes no change in gene expression (log2FC = 0). The extremely low correlation (R^2^ = 0.0001) indicates weak coupling between transcriptional and splicing regulation. (**C**) KEGG enrichment analysis of DEG-specific genes. (**D**) KEGG enrichment analysis of DASG-specific genes.

To further investigate the relationship between alternative splicing (AS) and transcriptional regulation, we compared differentially expressed genes (DEGs) and genes undergoing differential alternative splicing (DASGs) under light conditions at the early stage of salt stress (0 h vs. 2 h). Venn diagram analysis revealed that a large proportion of genes were uniquely regulated at either the transcriptional or splicing level. Specifically, 6573 genes were identified as DEGs only, while 1716 genes exhibited DAS events without significant changes in gene expression, whereas only 456 genes were shared between the two groups (Figure 6A). To further assess the relationship between gene expression and AS changes, we analyzed the correlation between log2 fold change (log2FC) and exon inclusion level difference (ΔPSI) for the overlapping genes. The results showed an extremely weak correlation (R^2^ = 0.0001), indicating no strong linear relationship between transcriptional and splicing changes (Figure 6B).

Functional enrichment analysis further revealed distinct biological roles for each gene category. The overlapping genes were mainly enriched in circadian rhythm, suggesting coordinated regulation at both transcriptional and splicing levels. Notably, the enrichment of circadian rhythm-related pathways among overlapping genes suggests a potential coordination between circadian regulation, transcription, and AS. In contrast, DEG-specific genes were predominantly enriched in metabolic pathways, including protein processing in the endoplasmic reticulum, biosynthesis of cofactor, biosynthesis of amino acid, and glutathione metabolism (Figure 6C), indicating that transcriptional regulation mainly contributes to metabolic reprogramming under salt stress. Meanwhile, DASG-specific genes were significantly enriched in RNA processing-related pathways, such as spliceosome and mRNA surveillance pathways (Figure 6D), suggesting that AS preferentially targets genes involved in post-transcriptional regulation and RNA metabolism. Taken together, these results indicate that alternative splicing and gene expression represent largely distinct but partially coordinated regulatory layers, contributing differentially to the early response to salt stress under light conditions.

### 2.6. DAS Events Within Light and Dark Conditions Separately

**Figure 7:**
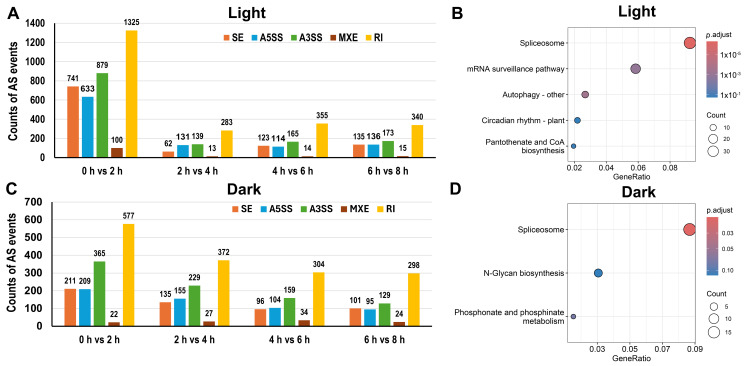
Differentially alternatively spliced events in light and dark conditions. (**A**) Number of differentially alternatively spliced events under light conditions. (**B**) KEGG enrichment of AS genes in light conditions (0 h vs. 2 h) under salt stress. (**C**) Number of differentially alternatively spliced events under dark conditions. (**D**) KEGG enrichment of AS genes in the dark group (0 h vs. 2 h) under salt stress.

We next examined DAS events within each light condition separately by comparing each post-stress time point to the corresponding 0 h baseline. In the SD condition, the 0 h vs. 2 h comparison yielded the largest number of DAS events across all pairwise comparisons, comprising 741 SE, 633 A5SS, 879 A3SS, 100 MXE, and 1325 RI events (Figure 7A, Table S4). This early burst of AS reprogramming was followed by a progressive decline in DAS event numbers at subsequent time points—283 RI events at 2 h vs. 4 h— before a partial recovery to 340 events at 6 h vs. 8 h. This temporal pattern indicates that the AS response to salt stress under light conditions is most dynamic during the initial stress phase, after which the splicing landscape transitions toward a relatively stable steady state.

KEGG enrichment analysis of DAS genes in the SD conditions at 0 h vs. 2 h revealed significant enrichment in spliceosome, mRNA surveillance pathway, Autophagy-other, Circadian rhythm—plant, and Pantothenate and CoA biosynthesis (Figure 7B, Table S5). Complementary GO enrichment analysis further revealed that these SD-specific early DAS genes were predominantly associated with RNA splicing, nuclear body, nuclear speck, mRNA processing, and regulation of alternative mRNA splicing via spliceosome, reflecting a broad reprogramming of RNA metabolism (Table S5). The prominent co-enrichment of spliceosome components and mRNA surveillance pathway genes indicates that the splicing machinery itself is subject to extensive post-transcriptional remodeling during the early salt stress response under light, suggesting a feedback regulatory mechanism by which AS regulates the activity of the very machinery that controls splicing.

In the SN condition, the 0 h vs. 2 h comparison yielded notably fewer DAS events compared to the corresponding SD comparison: 211 SE, 209 A5SS, 365 A3SS, 22 MXE, and 577 RI events (Figure 7C, Table S6). DAS event numbers remained comparatively moderate at subsequent time points, with 372 RI events at 2 h vs. 4 h, 304 RI at 4 h vs. 6 h, and 298 RI at 6 h vs. 8 h. KEGG enrichment of SN DAS genes at 0 h vs. 2 h identified spliceosome, N-Glycan biosynthesis, and phosphonate and phosphinate metabolism as the top enriched pathways (Figure 7D, Table S7). GO enrichment analysis of the SN DAS genes revealed a substantial overlap with SD in core RNA-related categories—including negative regulation of innate immune response, negative regulation of defense response, mRNA splicing via spliceosome, RNA splicing via transesterification reactions with bulged adenosine as nucleophile, and RNA splicing via transesterification reactions—underscoring the existence of a conserved post-transcriptional regulatory core activated by salt stress regardless of light conditions (Table S7). The shared enrichment of spliceosome pathway genes in both SD and SN conditions points to a conserved core AS regulatory program in response to salt stress that operates independently of light. By contrast, the unique enrichment of phosphonate and phosphinate metabolism and N-Glycan biosynthesis specifically in the SN conditions highlights condition-specific regulatory differences, reflecting the importance of metabolic energy state and transcriptional reprogramming when salt stress is experienced in the absence of photosynthetically generated energy.

### 2.7. Coordinated Expression of Spliceosome-Related Genes Under Light Conditions

**Figure 8:**
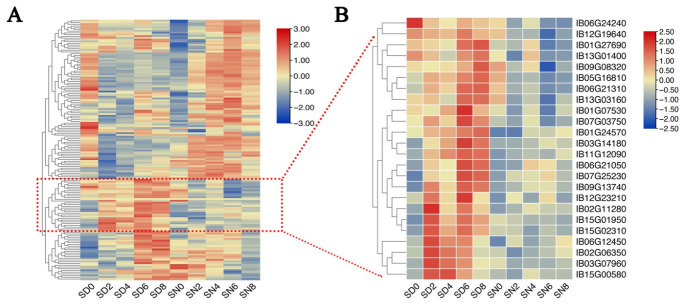
Coordinated expression patterns of spliceosome-related genes under light conditions. (**A**) Heatmap showing the expression profiles of spliceosome-related genes across all samples under light (SD) and dark (SN) conditions at different time points (0, 2, 4, 6, and 8 h). Rows represent genes and columns represent samples. Hierarchical clustering was performed to group genes with similar expression patterns. (**B**) Heatmap of selected spliceosome-associated genes with relatively high expression levels. Representative genes are shown to highlight key components of the splicing machinery during salt stress. The color scale indicates relative expression levels across samples.

To further explore the potential link between AS dynamics and the regulation of the splicing machinery, we examined the expression profiles of spliceosome-related genes across light (SD) and dark (SN) conditions (Figure 8A). The heatmap revealed a coordinated temporal pattern, with a substantial subset of spliceosome-associated genes showing relatively elevated expression during the early stages of salt stress (SD0–SD2), which coincides with the time window when DAS events are most abundant. This temporal concordance suggests that the rapid AS response observed under light conditions may be associated with a concurrent increase in the transcriptional activity of splicing-related genes. Notably, several key splicing regulators, including *RRC1*(*IB01G07530*), *CDC5*(*IB01G27690*), *SR45a*(*IB02G06350)*, *U2AF35B*(*IB03G07960*), *SRp34a*(*IB06G12450)*, and *SCL30A*(*IB12G23210*), displayed relatively high expression levels during the early stress phase (Figure 8B). These factors are known to participate in splice site recognition and spliceosome assembly, suggesting a potential role in modulating AS responsiveness under light conditions. In addition, other genes with relatively high expression levels, such as *THO2*(*IB12G19640*), *ELF5*(*IB15G00580*), and two SC35 homologs (*IB15G01950* and *IB15G02310*), further support the coordinated activation of RNA processing-related components. Taken together, these results indicate that light-associated AS dynamics may be accompanied by transcriptional modulation of spliceosome-related genes, pointing to a potential coordination between splicing regulation and gene expression during early salt stress responses.

### 2.8. Verification of AS Patterns in DAS Genes by RT-PCR

**Figure 9:**
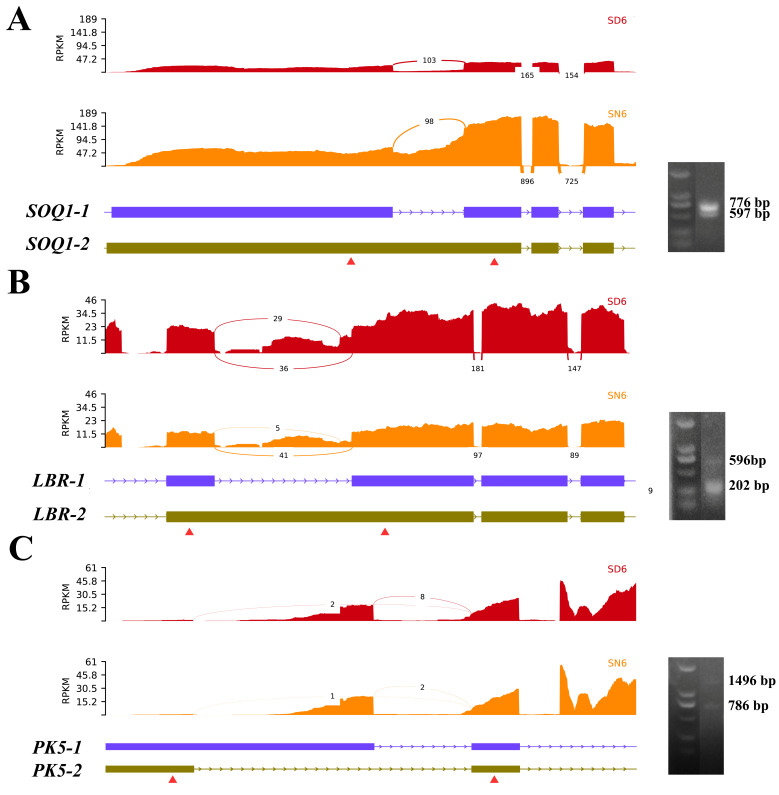
Experimental validation of alternative splicing events in representative DAS genes. (**A**–**C**) Sashimi plots showing RNA-seq read coverage and splice junction usage for three representative genes (SOQ1, LBR and PK5) under light (SD6) and dark (SN6) conditions. Arcs represent exon–exon junction reads, and numbers indicate supporting read counts. Gene structures are shown below each panel, with two major splice isoforms illustrated. Red triangles indicate the positions of primers used for semi-quantitative RT-PCR (sqRT-PCR). Corresponding agarose gel electrophoresis images are shown on the right, confirming the presence of distinct splice isoforms. Band sizes (bp) are indicated.

To validate the reliability of RNA-seq-based alternative splicing (AS) analysis, three representative DAS genes were selected for experimental verification using semi-quantitative RT-PCR (sqRT-PCR), with primers flanking the predicted AS regions (Figure 9). Sashimi plot visualization showed clear differences in read coverage and splice junction usage between light (SD6) and dark (SN6) conditions. These RNA-seq-based splicing patterns were further supported by sqRT-PCR results, which revealed distinct bands corresponding to different splice isoforms [29]. For the SOQ1 homolog (IB05G02580), two major splice isoforms (SOQ1-1 and SOQ1-2) were detected. The sashimi plots indicated intron retention between SD6 and SN6, which was consistent with the presence of two distinct PCR bands (~776 bp and ~597 bp), confirming condition-dependent isoform variation. Similarly, the LBR-1 homolog (IB01G01700) exhibited clear AS differences between light and dark conditions. The RNA-seq data suggested intron retention events, which were validated by sqRT-PCR, showing two isoforms (~596 bp and ~202 bp) with differential abundance between SD6 and SN6. For the PK5 homolog (IB01G01340), pronounced differences in transcript structure were observed, with differential exon usage between two conditions. The corresponding sqRT-PCR results confirmed two major isoforms (~1496 bp and ~786 bp), supporting the RNA-seq-inferred AS patterns. Overall, the high concordance between sashimi plots and RT-PCR results demonstrates the robustness of the RNA-seq-based AS identification. Notably, these genes are homologous to light-responsive or signaling-related genes in *Arabidopsis*, suggesting that light-modulated AS may contribute to functional regulation of stress and photoregulatory pathways under salt stress.

## 3. Discussion

Alternative splicing (AS) has emerged as a critical regulatory layer that enables plants to rapidly adjust gene expression in response to environmental fluctuations. In this study, we demonstrate that light conditions profoundly modulate salt stress-induced AS dynamics in sweet potato, not by altering the overall abundance of AS events, but by reshaping their temporal progression and functional specificity. Importantly, this modulation is manifested as a visible divergence between light and dark conditions, primarily reflected in distinct patterns of intron retention (IR). This finding highlights AS as a key interface integrating environmental signals, particularly light and salinity, into coordinated post-transcriptional regulatory programs.

**Figure 10:**
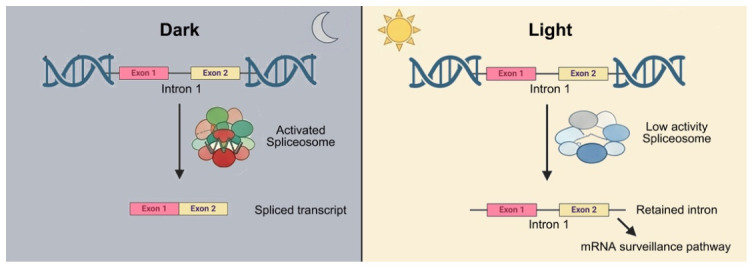
Proposed model of light-dependent regulation of intron retention and mRNA surveillance under salt stress in sweet potato. Under dark conditions, spliceosome activity remains relatively active, promoting efficient intron removal and the production of properly spliced transcripts. In contrast, under light conditions, spliceosome activity is relatively reduced or dynamically modulated, leading to increased intron retention (IR). These intron-retaining transcripts may contain premature termination codons (PTCs) and are subsequently recognized and targeted by the mRNA surveillance pathway, particularly nonsense-mediated decay (NMD). This coupling between IR and RNA quality control represents a regulatory mechanism through which light modulates transcriptome plasticity and stress adaptation.

A central observation of this study is the predominance of IR across two conditions, consistent with previous reports in diverse plant species, such as *Arabidopsis* [27], tea plant [30], and strawberry [26]. IR is widely recognized as the most prevalent AS type in plants and is often enhanced under stress conditions [31]. Rather than serving merely as a byproduct of inefficient splicing, accumulating evidence suggests that IR functions as a regulatory mechanism controlling transcript stability and translational potential. Notably, IR levels were higher under light conditions than under dark conditions, indicating that light promotes IR-associated regulatory processes during salt stress. Interestingly, despite this increase in IR, spliceosome-related genes were more strongly upregulated under dark conditions, suggesting that transcriptional activation of splicing factors does not necessarily correspond to reduced IR or enhanced splicing efficiency. Instead, this pattern indicates a regulatory decoupling between spliceosome gene expression and AS outcomes. In this context, elevated expression of splicing factors under dark conditions may represent a compensatory response to maintain basal splicing capacity, whereas light conditions may favor a more dynamic splicing environment that promotes regulated IR production. Importantly, IR-derived transcripts often contain premature termination codons (PTCs) and can be targeted by the mRNA surveillance pathway, particularly nonsense-mediated decay (NMD) [12,17]. Consistent with this, the enrichment of mRNA surveillance-related genes (Figure 7B) supports a coordinated regulatory mechanism rather than splicing noise. As illustrated in Figure 10, light-dependent modulation of AS promotes IR, which in turn channels transcripts into the mRNA surveillance pathway, forming a regulatory axis that controls transcript stability under stress conditions.

In contrast, other AS types, including exon skipping (ES), exhibited relatively minor changes between light and dark conditions and were not the primary contributors to the observed divergence. Although SE events were detected under both conditions (Figure 7A), their variation was limited compared with IR, suggesting that exon-level regulation plays a less dominant role in this context. This indicates that the light-dependent AS response in sweet potato is primarily driven by IR rather than by widespread changes in exon skipping [17]. Therefore, the increased IR frequency observed under light conditions may reflect enhanced transcriptional activity and more dynamic splice site selection. Importantly, our results further show that this shift is accompanied by coordinated transcriptional activation of spliceosome components. As evidenced by the expression heatmaps (Figure 8B), multiple core splicing regulators (e.g., SR proteins and spliceosome assembly factors) exhibit elevated expression during the early stages of salt stress under light conditions. This observation suggests that light not only influences transcriptional kinetics but may also enhance the capacity and responsiveness of the splicing machinery, thereby reinforcing AS responsiveness. This shift toward intron-level regulation implies that light not only promotes overall transcriptional activity but also refines transcript isoform diversity, potentially generating functionally distinct protein variants required for stress adaptation [32,33].

A particularly notable feature of the AS response is its temporal organization. Under light conditions, a pronounced burst of AS reprogramming was observed during the early phase of salt stress (0–2 h), followed by a gradual decline and partial stabilization at later stages (Figure 7A). This suggests that AS participates in both immediate stress perception and subsequent acclimation processes [34]. Notably, this early AS burst is largely attributable to a rapid increase in IR events under light conditions, further supporting the central role of IR in early stress responses. This early AS burst temporally coincides with the upregulation of spliceosome-related genes (Figure 6D), suggesting that transcriptional activation of splicing factors may contribute to the rapid reprogramming of AS under light conditions. In contrast, the AS response under dark conditions was more moderate and less dynamic, indicating a delayed or attenuated regulatory response. Future studies integrating AS profiling with physiological parameters, such as Na^+^/K^+^ homeostasis and reactive oxygen species (ROS) dynamics, will be essential to directly link splicing regulation with adaptive outcomes and strengthen causal inference beyond transcriptomic associations. These results collectively suggest that light enhances the responsiveness and plasticity of the AS regulatory network, enabling a more rapid reconfiguration of the transcriptome upon stress onset [35].

Functional enrichment analysis further revealed that light-dependent AS regulation is associated with two major functional modules: RNA processing and metabolic reprogramming. This observation further supports the mechanistic link between IR and RNA quality control, as depicted in Figure 10, where intron-retaining transcripts are preferentially targeted by the mRNA surveillance pathway. Recent studies have highlighted that reactive species (including ROS and RNS) function not only as damaging agents but also as central signaling molecules integrating multiple stress-response pathways [36]. In this context, reactive species-mediated signaling has been shown to interact with RNA processing pathways, including spliceosome activity and mRNA surveillance mechanisms. Given that these pathways are significantly enriched among the DASGs identified in our study, it is plausible that light-enhanced AS reprogramming may be coordinated with redox signaling networks to fine-tune stress-responsive gene expression. The enrichment of spliceosome components and mRNA surveillance pathway genes among early differentially alternatively spliced genes (DASGs) under light conditions suggests the existence of a feedback regulatory mechanism, whereby the splicing machinery itself is dynamically regulated through AS. Such autoregulatory loops have been well documented in serine/arginine-rich (SR) proteins, whose alternative splicing controls their own abundance and activity [37]. When combined with the observed upregulation of spliceosome-related genes (Figure 8B), these findings further support a self-reinforcing regulatory loop in which AS modulates the expression of splicing factors, which in turn reshape AS patterns, thereby amplifying the overall splicing response under light conditions. The concurrent enrichment of metabolic pathways, including fatty acid metabolism and terpenoid biosynthesis, indicates that AS may also contribute to stress adaptation by diversifying enzymes involved in membrane remodeling and secondary metabolite production [38,39]. This dual role of AS in maintaining transcriptome integrity and facilitating metabolic flexibility underscores its importance in coordinating complex stress responses.

Another key finding is the substantial divergence in differentially alternatively spliced genes (DASGs) between light and dark conditions, particularly at the intermediate stage of stress (6 h), where the highest number of DASGs was observed. This peak of divergence coincides with increased intron retention events and pronounced transcriptional differences, suggesting a coordinated regulation of AS and gene expression. The clustering analysis further supports this view, revealing distinct expression patterns between light- and dark-treated samples. Similar coupling of AS and transcriptional regulation has been described in tea plants, where differentially expressed AS genes in catechin biosynthesis pathways showed coordinated changes in both splicing pattern and transcript abundance in response to pathogen infection [31], highlighting a general principle by which AS and transcription work in concert to fine-tune stress-responsive gene output. In addition, the stronger induction of spliceosome-related gene expression under light conditions (Figure 8B) further suggests that differences in the regulatory capacity of the splicing machinery may contribute to the observed divergence in DASGs between light and dark environments. These observations indicate that AS regulation is not an isolated process but is tightly coupled with transcriptional control, collectively shaping the functional output of stress-responsive genes.

Based on these findings, we propose a working model (Figure 10) in which light modulates spliceosome activity and promotes intron retention (IR) during salt stress. In this model, increased IR generates transcripts that are selectively recognized by the mRNA surveillance pathway, thereby regulating transcript stability and gene expression outputs. Rather than representing splicing inefficiency, IR functions as a regulatory hub linking alternative splicing with RNA quality control. In contrast, under dark conditions, more efficient intron removal leads to the accumulation of fully spliced transcripts and a reduced reliance on RNA surveillance mechanisms. This model provides a conceptual framework for understanding how environmental signals converge at the post-transcriptional level to regulate plant stress responses.

Notably, previous studies in *Arabidopsis* have demonstrated that AS is tightly linked to circadian rhythms and exhibits diurnal patterns under light–dark cycles [40,41]. Whether the light–dark AS divergence observed here is conserved across different tissues or crop species remains unclear and warrants further investigation. From an applied perspective, the identification of light-responsive AS events and key DASGs provides valuable candidate targets for improving salt tolerance in sweet potato. In particular, the spliceosome-related genes identified in this study may represent important regulatory nodes for modulating AS efficiency and enhancing stress adaptability. Given that AS can generate multiple isoforms with distinct or even opposing functions, future studies should focus on the functional characterization of specific splice variants, particularly those associated with stress-responsive pathways. Integrating AS analysis with gene expression, protein function, and phenotypic data will be essential for translating these findings into molecular breeding strategies. In summary, our study demonstrates that light modulates salt stress-induced AS primarily by enhancing intron retention (IR) during the daytime, leading to a distinct temporal splicing pattern compared with nighttime conditions. By linking AS dynamics with the transcriptional regulation of splicing machinery components, this study provides new insights into how light coordinates transcriptional and post-transcriptional regulation during plant stress responses. In this study, selected AS events have been experimentally validated, and further isoform-specific validation will be performed to confirm their functional roles in future work. Furthermore, integrating alternative splicing analysis with proteomics and ribosome profiling will help to determine whether light-responsive splice isoforms lead to changes in protein abundance, translation efficiency, or subcellular localization under stress conditions.

## 4. Materials and Methods

### 4.1. Plant Growth Conditions and Treatments

To induce salt stress, the cultivar ‘Haida HD7791’ was used. Prior to treatment, sweet potato cuttings were surface-sterilized with a fungicide solution (1 g L^−1^ carbendazim for 5–8 min) and subsequently immersed in double-distilled water (ddH_2_O) until roots emerged. Once roots were well-established, seedlings were transplanted into containers (25 cm × 16.5 cm × 13.5 cm) filled with 4 L of half-strength Hoagland nutrient solution and maintained at 25–27 °C under a 12 h light/12 h dark photoperiod. Salt stress was imposed by supplementing the growth medium with 200 mM NaCl. Two light condition treatments were established: salt stress initiated during the daytime (designated as zeitgeber time 0 h), referred to as SD (Salt stress during the Day), and salt stress initiated during the nighttime (zeitgeber time 16 h), referred to as SN (Salt stress during the Night). After treatment initiation, plants were returned to the standard 12 h light/12 h dark photoperiod. Leaf tissues were harvested at 0, 2, 4, 6, and 8 h after the onset of salt treatment under each condition. All samples were immediately frozen in liquid nitrogen and stored at −80 °C until use. Each treatment included three independent biological replicates, with each replicate consisting of pooled leaf tissues from three individual plants.

### 4.2. RNA Extraction and RNA-Seq Library Construction

Total RNA was extracted from treated leaves using the Plant Tissue RNA Easy Fast Extraction Kit (Tiangen BiotechBeijing Co., Ltd., Beijing, China, #DP452) in accordance with the manufacturer’s instructions. The RNA concentration and purity were measured using a NanoDrop 2000 spectrophotometer (Thermo Fisher Scientific, Wilmington, DE, USA), while RNA integrity was evaluated with the RNA Nano 6000 Assay Kit on the Agilent Bioanalyzer 2100 system (Agilent Technologies, Santa Clara, CA, USA). Only RNA samples with an RNA Integrity Number (RIN) ≥ 9.0 and an OD260/280 ratio between 1.8 and 2.0 were selected for library construction. Poly(A)-enriched mRNA libraries were prepared using the NEBNext Ultra RNA Library Prep Kit for Illumina (NEB, Ipswich, MA, USA) following the manufacturer’s protocol. Sequencing was then performed on an Illumina HiSeq 4000 platform, yielding 150 bp paired-end reads.

### 4.3. RNA-Seq Data Processing

Raw sequencing reads were quality-filtered using fastp (v0.23.4) [42] to remove adapter sequences and low-quality bases (quality score < 20). The clean reads were then aligned to the sweet potato reference genome (*Ipomoea batatas* cv. Taizhong 6, v1.0.a2 annotation) [43,44] using STAR (v2.7.11b) in two-pass mode with default parameters. Transcript assembly and quantification were subsequently performed using StringTie (v2.1.5) [45] following default parameters. The resulting transcripts (GTF files) for 30 libraries were then merged using the multi-sample transcriptome assembly tools named TACO (v0.7.3) [46]. Differential gene expression analysis was performed using the DESeq2 R package (v1.16.1) [47]. Genes with a false discovery rate (FDR) < 0.01 and |log2-fold change| > 1 were considered differentially expressed genes (DEGs). Finally, gene and transcript expression levels were estimated as Transcripts Per Million (TPM) values using Salmon v1.10.0 [48].

### 4.4. Identification and Characterization of AS Events

To classify the AS events, the tool AStalavistav4.0 [49] was employed using the raw .gtf file assembled from transcriptome data. Four major types of AS events were identified: intron retention (IR, AS code: 1^2-, 0), exon skipping (ES, AS code: 1-2^, 0), alternative 3′ splice site (A3S, AS code: 1-, 2-), and alternative 5′ splice site (A5S, AS code: 1^, 2^). These AS events were extracted from the output files and quantified. Statistical analyses of AS event counts were performed across all time points under both light and dark conditions. Additionally, the density distribution of AS genes across chromosomes was visualized. In this study, DASGs were defined as genes containing one or more differential alternative splicing (DAS) events. When summarizing at the gene level, DASGs were categorized based on the predominant direction of change in exon inclusion levels (IncLevel) of their associated DAS events, rather than overall gene expression levels.

### 4.5. Identification of Differentially Alternatively Spliced (DAS) Events

Differential alternative splicing (AS) analysis was conducted using rMATS-turbo (v4.3.0) [28] by comparing treatment and control groups, with aligned BAM files as input with default settings applied. For each pairwise comparison, rMATS-turbo calculated the percentage spliced in (IncLevel) for each sample and identified significant differences in IncLevel between treatment and control groups. DAS events were filtered based on two criteria: an absolute IncLevelDifference > 0.1 and a false discovery rate (FDR) < 0.05. Additionally, differential AS analysis was performed between light and dark conditions at corresponding time points to identify splicing changes specific to each light condition.

### 4.6. Functional Enrichment Analysis

GO and KEGG pathway enrichment analyses were carried out for genes containing DAS events using the clusterProfiler v4.20.0 software [50]. Enrichment significance was determined using the hypergeometric test, with results considered significant at a corrected *p*-value < 0.05. Venn diagrams were constructed to identify common and unique DAS genes between light and dark conditions across different time points.

### 4.7. Validation of AS-Events RT-PCR

The Total RNA was extracted and reverse-transcribed into first-strand cDNA using a PrimeScript RT Reagent Kit (Takara, Otsu, Japan) following the manufacturer’s instructions. Alternative splicing (AS) events were identified from RNA-seq data and visualized using Integrative Genomics Viewer (IGV), and sashimi plots were generated to illustrate splice junction usage. Gene-specific primers were designed to flank the alternative splicing regions based on predicted isoforms. PCR products were separated on 1.5–2.0% agarose gels and visualized under UV light. Different band sizes corresponding to distinct splice isoforms were used to assess AS patterns.

## 5. Conclusions

In summary, our study demonstrates that light does not markedly change the overall number of AS events but leads to a visible shift in AS patterns, primarily through enhanced intron retention (IR) under light conditions. This results in distinct splicing responses between daytime and nighttime salt stress conditions. Future experimental validation of key AS events will further clarify their roles in stress adaptation. These findings highlight AS as a key regulatory interface integrating environmental signals and provide a foundation for future functional studies of splicing-mediated stress adaptation in sweet potato.

## Data Availability

The original data presented in the study are openly available in the Genome Sequence Archive at https://ngdc.cncb.ac.cn/search/ (assessed on 1 May 2026) under accession number PRJCA062955.

## Supplementary Materials

The following supporting information can be downloaded at: https://www.mdpi.com/article/10.3390/plants15101556/s1, Table S1. Summary of RNA-seq read statistics; Table S2. GO and KEGG enrichment analysis of AS genes between light and dark groups; Table S3. Differentially alternatively spliced (AS) events between light and dark groups under salt stress at different time points; Table S4. Differentially alternatively spliced (AS) events in the light group under salt stress at different time points; Table S5. GO and KEGG enrichment analysis of AS genes in the early light condition (0 h vs. 2 h) under salt stress; Table S6. Differentially alternatively spliced (AS) events in the dark group under salt stress at different time points; Table S7. GO and KEGG enrichment analysis of AS genes in the early dark condition (0 h vs. 2 h) under salt stress. Table S8. Primers used in RT-PCR verification.
